# Functional Adrenocortical Carcinoma: A Rare Case With Thrombus Extension Into the Inferior Vena Cava and a Presentation of Cushing Syndrome

**DOI:** 10.7759/cureus.14239

**Published:** 2021-04-01

**Authors:** Thomas M Southall, Morgan MacDonald, Matthew R Acker, Michael Organ

**Affiliations:** 1 Faculty of Medicine, Memorial University of Newfoundland, St. John's, CAN; 2 Department of Urology, Dalhousie University, Halifax, CAN

**Keywords:** adrenocortical carcinoma, thrombus, cushing syndrome

## Abstract

Adrenocortical carcinoma (ACC) is a rare, highly malignant endocrine tumor, often associated with a poor prognosis. Most patients who develop ACC are either children of ages 1-6, or adults in their fourth to fifth decade of life. Individuals with a functional cortisol-secreting ACC frequently present with Cushing syndrome. We report a case of an 18-year-old male who was found to have a large ACC tumor, with thrombus extension into the inferior vena cava (IVC), after presenting with Cushing syndrome. ACC presents a challenging scenario for physicians as surgical resection remains the only form of curative therapy, however, despite such treatment many patients quickly develop metastases.

## Introduction

Adrenocortical carcinoma (ACC) is a rare, highly malignant endocrine tumor arising from the adrenal cortex, with an annual incidence of one per million per year [1]. ACC can develop at any age but typically presents in a bimodal distribution, either in childhood (1-6 years old) or in the fourth to fifth decade of life [2]. The prognosis is generally poor, with five-year overall survival of less than 50%, and less than 20% in those with metastatic disease [1]. Patients with ACC frequently present with Cushing syndrome, which can include truncal obesity, diabetes, hypertension, easy bruising, and virilization [3]. Recent systematic reviews and meta-analyses show a higher mortality and recurrence risk for cortisol-secreting ACCs, however, it remains unclear whether this association is due to the negative effects of cortisol, whether cortisol-secreting ACCs are simply a more aggressive subtype, or whether the cortisol is an independent prognostic marker [4]. Effective management usually involves care from a multidisciplinary team, with surgical resection representing the only curative treatment option [5]. Unfortunately, despite complete surgical resection, nearly 80% of patients will develop local or distant recurrence [2].

## Case presentation

**Figure 1:**
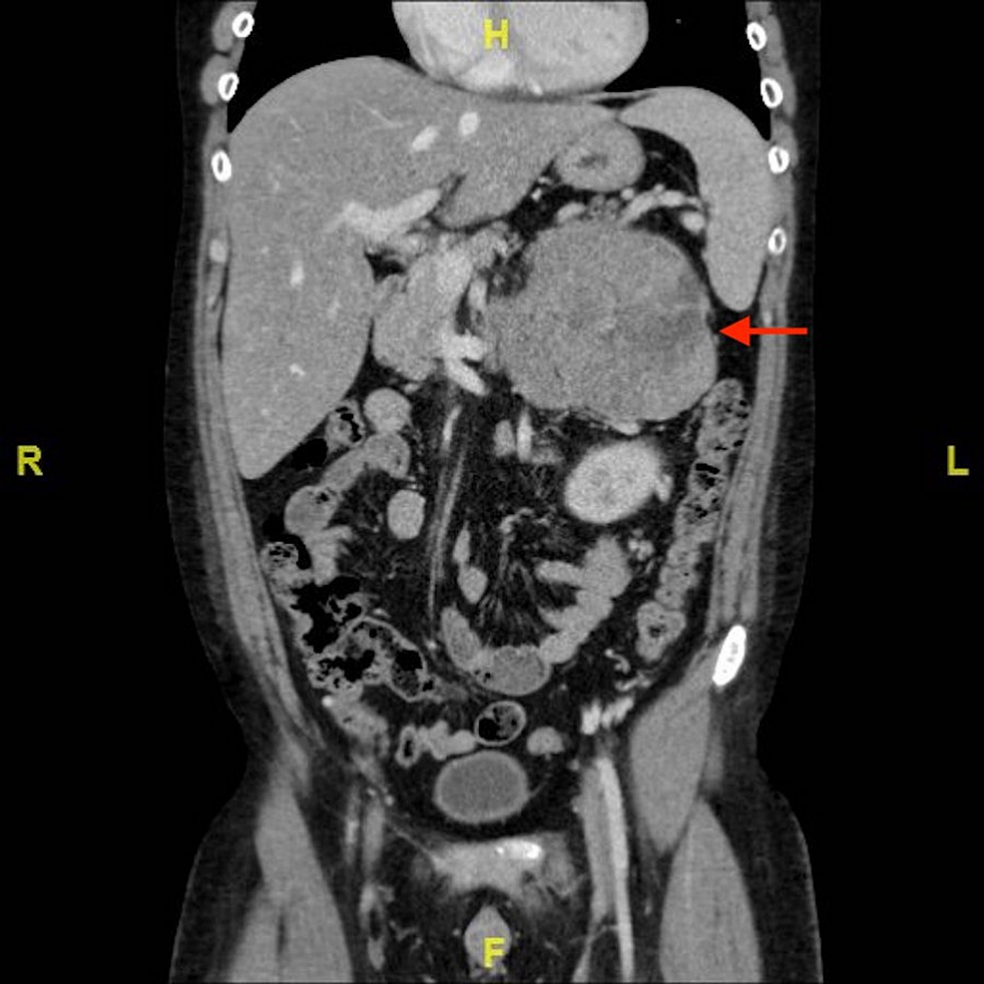
CT scan shows a large heterogeneous mass in the area of the left adrenal gland (red arrow), with numerous collateral vessels.

**Figure 2:**
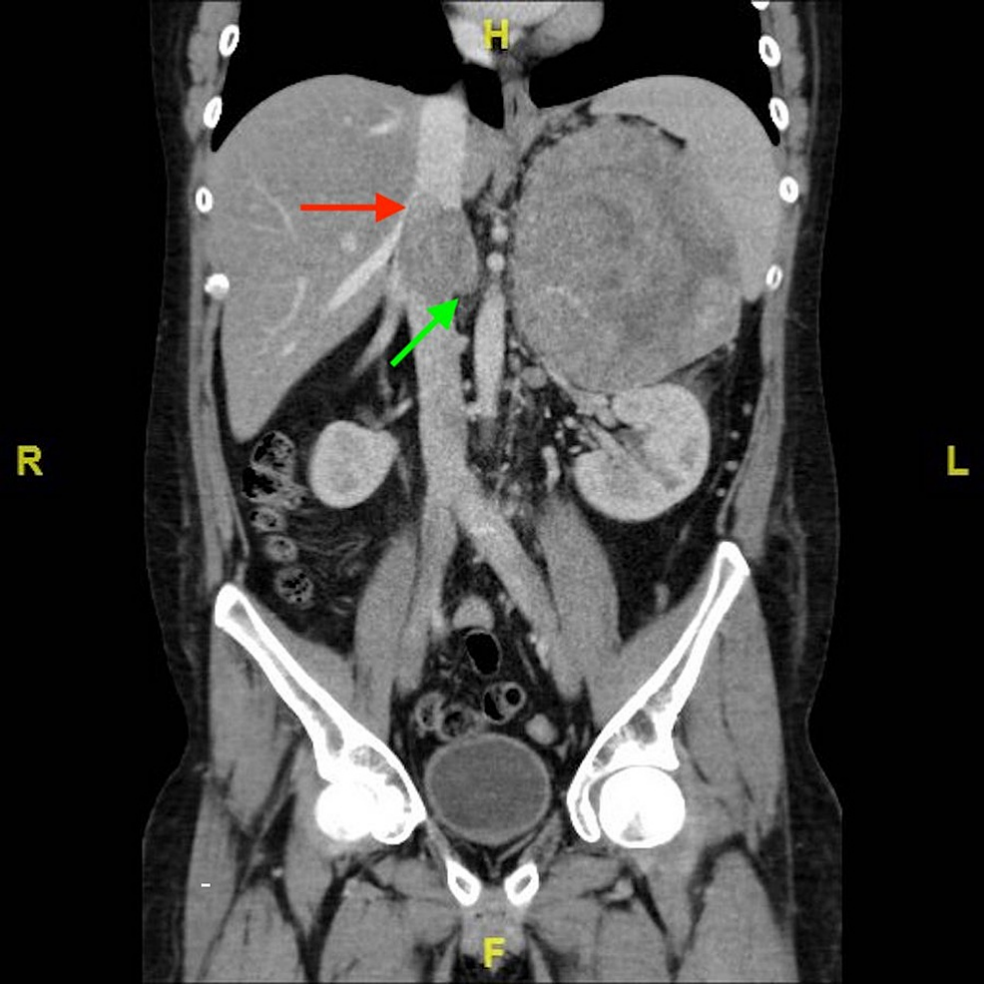
CT scan showing tumor thrombus extending into IVC (red arrow) and left renal vein (green arrow). IVC: Inferior vena cava.

**Figure 3:**
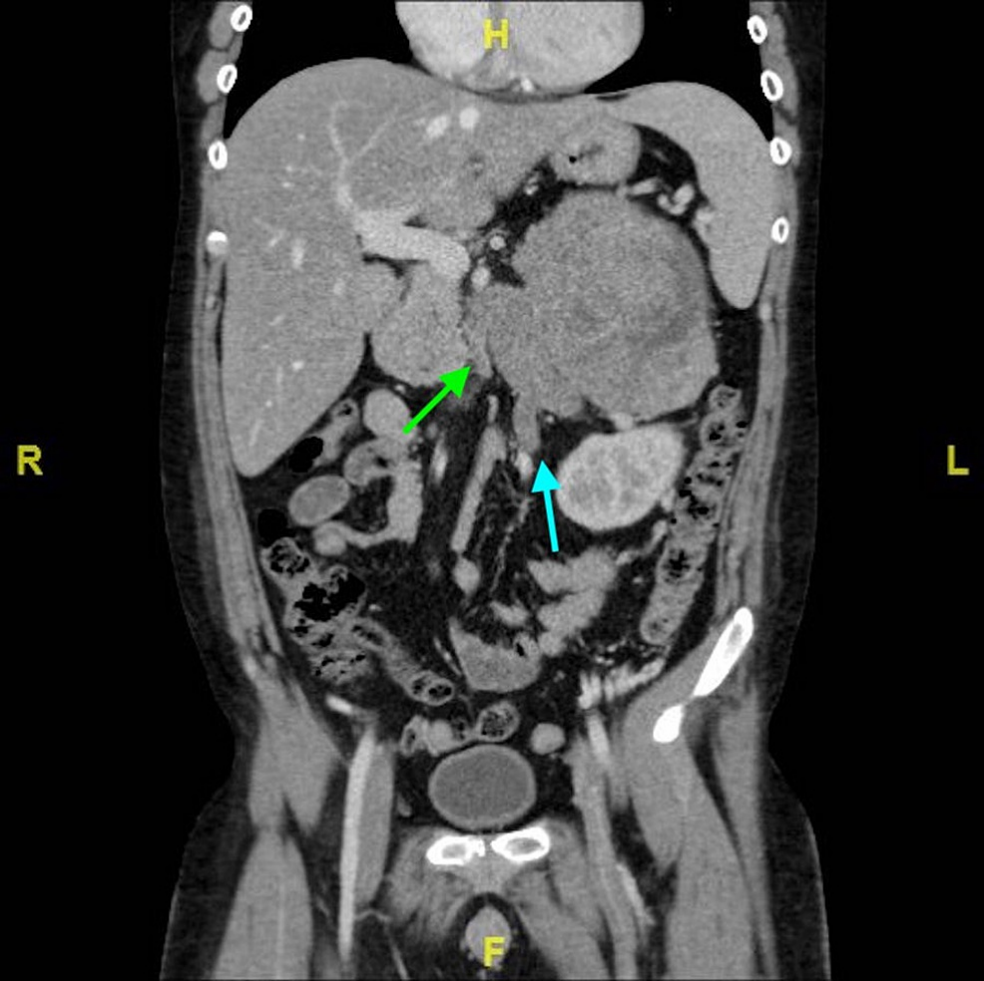
CT scan showing tumor thrombus extending into a left renal vein (green arrow) and left gonadal vein (blue arrow).

An 18-year-old male was referred to our hospital for further evaluation of lower extremity swelling. He had also developed new acneic skin issues, hypertension, truncal obesity, temporal fullness, thickening of facial hair, and skin striae involving the axillae, groin, and lower abdomen. A diagnosis of Cushing syndrome was made after serum cortisol came back elevated with adrenocorticotropic hormone (ACTH) being decreased. In Figure 1, a coronal section from his CT with contrast displays a large heterogeneous mass in the area of the left adrenal gland, measuring 14.3 x 10.7 x 13.6 cm. In Figure 2, it can be appreciated that the mass is surrounded by a significant number of collateral vessels, with tumor thrombus extending into the left renal vein, gonadal vein (Figure 3), and the inferior vena cava (IVC) at the intrahepatic level. Collateral vessels can also be seen around the kidney. No evidence of metastasis was identified. The primary lesion was suspicious for ACC.

An open left radical adrenalectomy and nephrectomy with IVC thrombectomy were performed. Final pathology was consistent with ACC of the left adrenal gland demonstrated by a Weiss score of 6. The specimen included vascular invasion, caval thrombus, and 11 interaortal caval lymph nodes negative for malignancy.

The patient recovered well from surgery. He subsequently received adjuvant radiation. While being considered for mitotane therapy, the patient underwent repeat CT imaging three months postoperatively and was found to have developed liver and distant metastases. After thorough consideration, he declined palliative treatment and succumbed to his disease shortly thereafter.

## Discussion

ACCs are devastating tumors that continue to burden patients with a very poor prognosis. Physicians currently lack a strong method for early detection of these tumors, which may partially explain why most ACC tumors are identified in advanced stages [6]. The majority of patients who develop ACC are females (57.2%) who present with a primary malignancy and unilateral adrenal involvement [7]. ACC occurs slightly more frequently on the left side (56.4%), however, the reason for this difference remains elusive [6]. Approximately 20% of patients present with at least one additional malignancy, which is believed to be largely due to the association between ACC and many hereditary conditions, such as Beckwith-Wiedemann syndrome and Li-Fraumeni syndrome [8].

ACC can present as either a functional or non-functional tumor, with each accounting for approximately half of all cases [4]. Isolated cortisol-secreting tumors, similar to the one presented in this case, are the most common functional subtype of ACC [1]. Additional subtypes include mixed hormone-secreting (25%), isolated sex hormone-secreting (20%), and isolated aldosterone-secreting (7.9%) tumors [6]. Patients with functional ACC tumors often present with symptoms secondary to excessive hormone secretion, such as Cushing syndrome secondary to excess cortisol secretion demonstrated in this case. Patients with non-functional ACC often present with complaints of abdominal or flank pain due to mass effect [6]. Another presentation that is becoming more common with current advances in healthcare is the incidental discovery of the tumor on imaging done for unrelated reasons [7]. The final diagnosis relies on pathological assessment and use of the Weiss score [9]. Scoring is based on evaluation of cellular architecture, the nucleus, and observed invasion, where scores ≥3 indicate ACC [10].

Surgical resection remains the only curative treatment option for ACC [5]. Adjuvant therapies often include local radiation therapy or adjuvant chemotherapy consisting of mitotane alone, mitotane with other systematic chemotherapy, or multi-agent chemotherapy without mitotane [6]. Unfortunately, despite the addition of such treatments, a recent population-based study of ACC found the median survival time of 14 months for patients diagnosed with ACC between 2005 and 2014 [7]. Given that many patients present with advanced disease, often with thrombus extension into the IVC, management requires multidisciplinary coordination, comprehensive pre-operative evaluation, and detailed surgical planning [5].

## Conclusions

This case demonstrates a rare case of cortisol-secreting ACC with invasion into the IVC. ACC is an aggressively malignant tumor with surgical resection as the only curative option. A substantial subset of patients who develop ACC present with Cushing syndrome. Due to the aggressive nature of ACC, it is important for physicians to be aware of such presentations so that early recognition and treatment can begin. Despite resection, the large majority of patients go on to develop metastatic disease. These tumors present a challenging scenario for physicians as current literature does not offer great insight into options for the prevention or prediction of metastasis.
